# High‐resolution structures of transient receptor potential vanilloid channels: Unveiling a functionally diverse group of ion channels

**DOI:** 10.1002/pro.3861

**Published:** 2020-04-11

**Authors:** Mark K. van Goor, Leanne de Jager, Yifan Cheng, Jenny van der Wijst

**Affiliations:** ^1^ Department of Physiology, Radboud Institute for Molecular Life Sciences Radboud University Medical Center Nijmegen The Netherlands; ^2^ Department of Biochemistry and Biophysics University of California San Francisco San Francisco California United States; ^3^ Howard Hughes Medical Institute University of California San Francisco California United States

**Keywords:** channel gating, cryo‐EM, selectivity, structure, TRP channels

## Abstract

Transient receptor potential vanilloid (TRPV) channels are part of the superfamily of TRP ion channels and play important roles in widespread physiological processes including both neuronal and non‐neuronal pathways. Various diseases such as skeletal abnormalities, chronic pain, and cancer are associated with dysfunction of a TRPV channel. In order to obtain full understanding of disease pathogenesis and create opportunities for therapeutic intervention, it is essential to unravel how these channels function at a molecular level. In the past decade, incredible progress has been made in biochemical sample preparation of large membrane proteins and structural biology techniques, including cryo‐electron microscopy. This has resulted in high resolution structures of all TRPV channels, which has provided novel insights into the molecular mechanisms of channel gating and regulation that will be summarized in this review.

## 1 INTRODUCTION

The transient receptor potential (TRP) superfamily is the second largest class of ion channels. There are six TRP channel subfamilies in mammals, the canonical (TRPC), melastatin (TRPM), vanilloid (TRPV), polycystin (TRPP), ankyrin (TRPA), and mucolipin (TRPML) channels, for a total of 28 mammalian family members.^1^ Most TRP channels are relatively nonselective cation (mainly calcium) channels. They are distantly related to voltage‐gated ion channels (VGICs), a group of ion channels that share a voltage‐dependent activation mechanism. TRP channels, on the other hand, have evolved to respond to a more diverse set of stimuli. Remarkably, single TRP channel members are often able to sense multiple environmental factors and are therefore considered polymodal sensors of the environment. They are involved in numerous biological processes in virtually all human tissues, ranging from calcium reabsorption in the kidney to the signal transduction pathways required for axon cone guidance in the brain.^2^, ^3^, ^4^ Perhaps their most well‐known function, however, is in sensory neurons, where they act as signal transducers by probing extracellular temperature, ligands, lipids, pH, and membrane potential.^2^ Disturbed TRP channel activity is increasingly being associated with a wide range of pathologies, including skeletal dysplasias, respiratory diseases, several types of cancer and diseases that affect the nervous system.^5^ Due to their role in pathophysiology and their localization at the plasma membrane, several members of the TRP channel superfamily are considered as promising drug targets.^6^ However, the development of efficient drugs targeting TRP channels has been slow, partly due to the fact that there were no high‐resolution TRP channel structures available.

Traditionally, high‐resolution structure determination of integral membrane proteins relies on X‐ray crystallography. TRP channels, however, resisted crystallization attempts and only in 2013, after technological advances in single particle cryogenic‐electron microscopy (cryo‐EM), the first TRP channel structure was determined.^7^, ^8^ This structure of TRPV1 marked the beginning of an impressive structural biology revolution for TRP channels, aided by a rapidly expanding cryo‐EM field.^9^ As of 2019, atomic structures of the majority of TRP channels are determined, and specifically for all TRPV subfamily channels. Almost all of these structures were determined by single particle cryo‐EM, with TRPV6 and TRPV4 as the only exceptions.^10^, ^11^ While the resolutions of these two crystal structures are no better than the corresponding cryo‐EM structures of the same channel, they do provide unambiguous identity of bound ions along the ion permeation pathway. An exciting prospect is that, with the rapid technological developments, single particle cryo‐EM will likely be able to reveal protein dynamics, providing more in‐depth insights into the dynamic process of channel regulation by ligands or other physiological stimuli. Even as it stands now, the insights gained from the recently published TRPV structures, combined with those obtained in functional and pharmacological studies of TRPV channel activity and modulation, are rapidly bringing the TRPV subfamily structure–function relationship into view.

With this review we aim to outline what the recent structural revolution has already meant for TRPV biology and, in particular, how it has impacted our understanding of the TRPV structure–function relationship. The structures will be used as a framework to discuss the most recent models for TRPV channel gating and how they are regulated by lipids and ligands. Finally, we explore some of the remaining challenges in TRPV channel biology.

## 2 TRPV CHANNEL STRUCTURE

**FIGURE 1:**
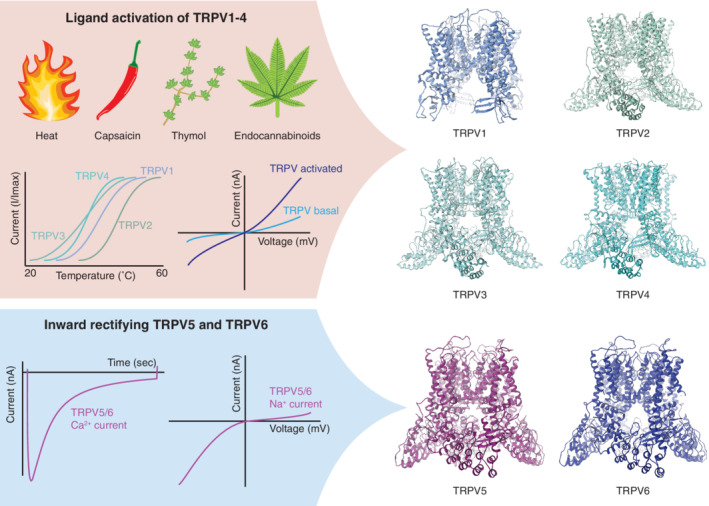
Schematic representation of the functional characteristics of the transient receptor potential vanilloid (TRPV) family members. The TRPV family is subdivided into the thermo‐sensitive and ligand‐activated TRPV1–4 and the calcium‐selective TRPV5/6. Structures of the different TRPV members are shown on the right (PDBs in order from TRPV1 to TRPV6: 5IRX, 5AN8, 6DVW, 6BBJ, and 6O20). The upper panel (in pink) depicts several natural stimuli of TRPV1‐4, as well as a schematic overview of relative thermosensitivity, and their current–voltage relationship of channel activity at basal and activated state. The bottom panel (in blue) shows the typical calcium current and subsequent channel inactivation, as well as the current–voltage relationship of TRPV5/6

The TRPV subfamily consist of six members, which can be further categorized into two rather distinct groups, based on functional characteristics. TRPV1–4 are fairly nonselective cation channels that are gated by physical or chemical stimuli, and are involved in sensory processes, such as thermosensation, chemosensation, and nociception^2^ (Figure 1). TRPV5 and TRPV6, on the other hand, are epithelial channels that are highly selective for calcium and facilitate calcium influx in kidney and intestine, important for the maintenance of calcium homeostasis.^3^, ^12^ They are likely not ligand‐gated^13^ and are constitutively active under normal physiological conditions, where they conduct almost exclusively calcium ions. They also permeate monovalent cations in the absence of calcium, which is frequently used in experimental settings.^13^ A functional hallmark of TRPV5/6 channels is their calcium‐dependent inactivation mechanism, which follows a negative feedback loop of increased intracellular calcium concentration and binding by the calcium‐sensing protein calmodulin (CaM)^14^, ^15^ (Figure 1).

**FIGURE 2:**
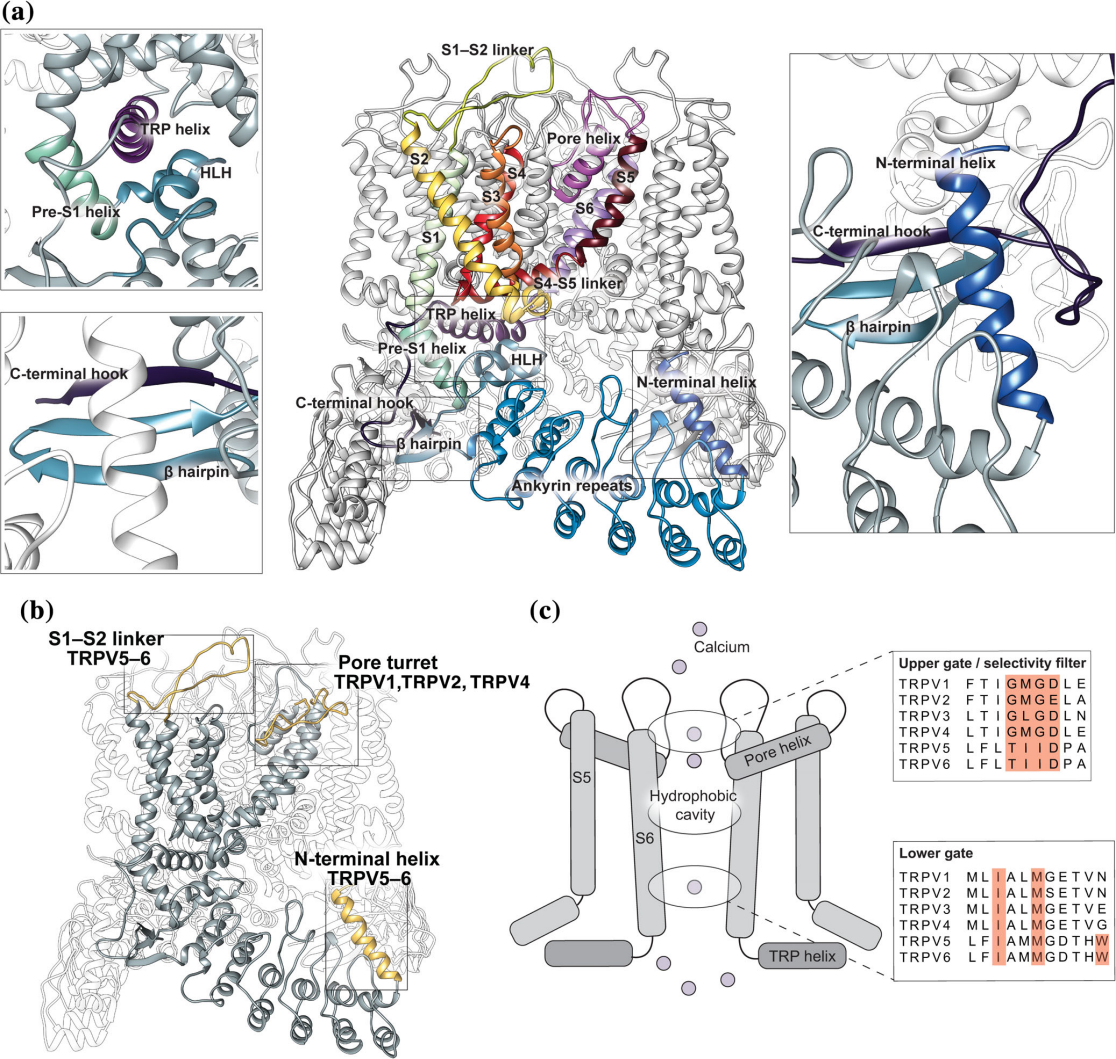
Canonical transient receptor potential vanilloid (TRPV) channel architecture. (a) Side view of TRPV5 channel (PDB: 6O1P). Different domains are colored separately. The boxes zoom in on relevant interactions between different domains. Relevant domains are colored and named analogous to the overview. (b) Side view of a chimeric TRPV structure, generated using the structural data of rTRPV2 (PDB: 6BO4) and rbTRPV5 (PDB: 6O1P). Unique domains are highlighted in yellow, related names and family members are mentioned. (c) Schematic representation of the ion permeation pathway of a TRPV channel, with a sequence alignment of the upper and lower pore of all human members. Residues important for restriction and/or channel gating are highlighted in red

Remarkably, despite being functionally very different, all TRPV channel structures share a highly conserved six transmembrane domain architecture, reminiscent of VGICs.^8^, ^10^, ^16^, ^17^, ^18^, ^19^, ^20^, ^21^, ^22^ Functional TRPV channels are tetrameric assemblies that surround a central permeation pathway. Individual subunits contain six transmembrane helical segments (S1‐6) with large intracellular amino (N) and carboxyl (C) termini (Figure 2). Like most VGICs and all other TRP channels with resolved structures, the transmembrane domain of TRPV channels has a domain‐swapped architecture, where the pore region (S5–S6) of one subunit is packed against the S1–S4 bundle of a neighboring subunit. A bulky intracellular skirt, dominated by characteristic N‐terminal ankyrin repeat domains (ARDs), hangs below the transmembrane domain and forms an intracellular vestibule. Located in the cracks between ARDs of neighboring subunits are interaction domains that facilitate channel assembly from single subunits (Figure 2a, insets). Yet, certain domains are unique to some members, like the S1‐S2 linker for TRPV5–6 and the pore turret for TRPV1, TRPV2, and TRPV4 (Figure 2b).

The overall architecture of the ion permeation pathway is highly similar among all TRPV channels, with the pore formed by S5 and S6 with an intervening short pore loop and helix (S5‐P‐S6). Along the pore, there are two main restriction points that gate the channel (Figure 2c). The first restriction point is at the upper part of the pore, typically comprised of a four amino acid stretch, including at least one negatively charged amino acid. This is also where the selectivity filter is located. The negative charge recruits and coordinates cations and the conformation of the selectivity filter determines the ion selectivity profile. The thermosensitive TRPV1–4 channels have a selectivity filter that is comprised of a GxGD/E motif (with the X being a lysine or methionine), while the highly calcium‐selective TRPV5 and TRPV6 channels have evolved with a distinct TIID motif (Figure 2c). The second restriction point is located near the helical bundle crossing of S6 at the bottom of pore, where either an isoleucine (TRPV1), a methionine (TRPV2, TRPV3, TRPV4, and TRPV6), or both (TRPV5) residues have been assigned to the lower restriction point (Figure 2c). In addition, TRPV5 and TRPV6 have a ring of tryptophan residues at the bottom of their pore, which plays an important role in the unique gating mechanism of these channels.

## 3 CHANNEL GATING

Obtaining structural insight into how TRPV channels are gated, requires high‐resolution structures of the channels at different gating states. Conformations of closed channels are often obtained from apo structures, or in some cases from structures of channels with an antagonist bound, which stabilizes a closed conformation. Structures with an open channel are, however, harder to obtain, except only in a few cases. So far, all structures of open channels were determined with one or more ligands bound, such as the structure of TRPV1 in complex with RTX and DkTx.^23^

### 3.1 *Selectivity versus flexibility*

For TRPV1, DkTx binding to the outer pore region causes a major conformational change of the upper pore region that reorients the outer pore loop and widens the upper pore.^23^ Another example of upper pore movement was recently demonstrated in the structures of a mutant TRPV2 channel, which had vanilloid‐sensitivity engineered into its sequence, based on findings from previous studies.^24^, ^25^, ^26^ The channel structures were resolved, bound by RTX, in an open conformation with a novel pore arrangement.^25^, ^27^ RTX binding elicited a pore widening that enabled permeation of molecules larger than cations and demonstrated significant uptake of a ~0.3 kDa fluorescent molecule (YO‐PRO‐1) upon stimulation with RTX.^25^ This phenomenon has previously been referred to as pore dilation, a unique property of TRPV1 and TRPV2, which occurs during sustained activation.^28^, ^29^ In addition, Zubcevic et al. showed that pore dilation in vanilloid‐sensitive TRPV2 is likely the cause of asymmetrical widening of the selectivity filter and they postulate that this unique plasticity is determined by large conformational changes of the S4‐S5 linker.^25^, ^27^ Asymmetric rearrangement of the selectivity filter was also seen in recent molecular dynamic simulation studies of TRPV1 and in voltage‐gated sodium channels.^30^, ^31^, ^32^, ^33^ Taken together, asymmetric pore configurations may be required for some forms of thermosensation and/or ligand activation in TRPV1 and TRPV2. However, so far, no such rearrangement has been observed in the other TRPV channels.

The conformation of the selectivity filter in TRPV5 and TRPV6 is highly similar in all resolved structures.^21^, ^22^, ^34^, ^35^, ^36^, ^37^ In contrast to the flexibility of the TRPV1 and TRPV2 selectivity filters, TRPV5/6 likely need a static selectivity filter to ensure their calcium‐conducting nature with high selectivity.^22^, ^35^, ^36^ Such rigidity of the upper pore resembles the upper pores of potassium‐selective VGICs which are also thought to move only minimally during channel activation, thereby maintaining a strict selectivity for potassium over sodium.^38^ It has been postulated that the rigidity of the TRPV5 and TRPV6 upper pore is due to a ring of phenylalanine residues in the pore helix, which restrict flexibility of surrounding residues.^11^ Furthermore, an extended S1‐S2 linker, unique to TRPV5 and TRPV6, folds back on top of the upper pore region. This results in a tight web of interactions with the pore loop. Thereby, it likely shields the upper pore of TRPV5 and TRPV6 from binding extracellular allosteric modulators and restricts flexibility of residues in the outer vestibule and selectivity filter.^11^, ^36^

### 3.2 *Movement of the lower gate*

Opening of the lower gate in response to agonist binding is primarily associated with movement of the S6 helix. Although, the exact movement of the S6 helix differs per channel subtype and is dependent on the agonist(s) that were used to activate the channel. In any case, activating movements in the S6 helix have in common that they widen the lower restriction site to a diameter that is sufficient for cations to pass through. One type of S6 movement is proposed to be associated with a S6 helical register shift.^17^, ^18^, ^19^, ^22^, ^35^, ^39^ In the closed structures of TRPV2,^16^, ^17^, ^25^ TRPV3,^18^, ^19^ and TRPV6,^22^ S6 has an α‐helical conformation, whereas in the “open” TRPV3 and TRPV6 structures S6 adopts a π‐helical conformation.^19^, ^22^ The effect of an α‐to‐π helical register shift is that downstream residues rotate along the helical axis, which changes the pore‐lining residues. It has been suggested that this transition represents a conserved mechanism of TRPV lower gate opening, by exposing a larger number of hydrophilic and negatively charged residues to the pore. Remarkably, the α‐to‐π transition is not dependent on sequence, since the residue that marks the register shift differs between TRPV channels. However, we doubt that an α‐to‐π transition of S6 is a general mechanism of opening or closing of the TRPV lower gate, since it was not observed in the structures of TRPV1 and TRPV5.^23^, ^36^

Most TRPV channels are polymodal, in which the upper and lower gates are targeted separately by different ligands. The upper and lower gates are proposed to be coupled allosterically so that opening of one gate, either the upper or lower one, would lead to the opening of the other. However, it remains unclear how and under what structural conformations coupling of the two gates occurs.

## 4 CHANNEL REGULATION

Structural studies of TRPV channels not only provided more insight into channel gating, they also broadened our understanding of channel modulation by exogenous and endogenous ligands or cofactors. Structural studies of TRPV channels reconstituted into lipid nanodiscs, also enabled visualization of many specific lipid protein interactions. Some of these observed interactions have significant functional roles, but others may not be physiologically relevant and may have been introduced during lipid nanodisc reconstitution. One particularly interesting observation is that in the apo state, ligand binding pockets are often occupied by endogenous lipids. Moreover, the recent structures of TRPV5 and TRPV6 in complex with CaM have shed new light on protein‐dependent TRPV channel regulation, characterizing a calcium‐dependent channel inactivation mechanism that is dependent on concerted movements of both the channel and CaM.

### 4.1 *Lipid and ligand binding*

**FIGURE 3:**
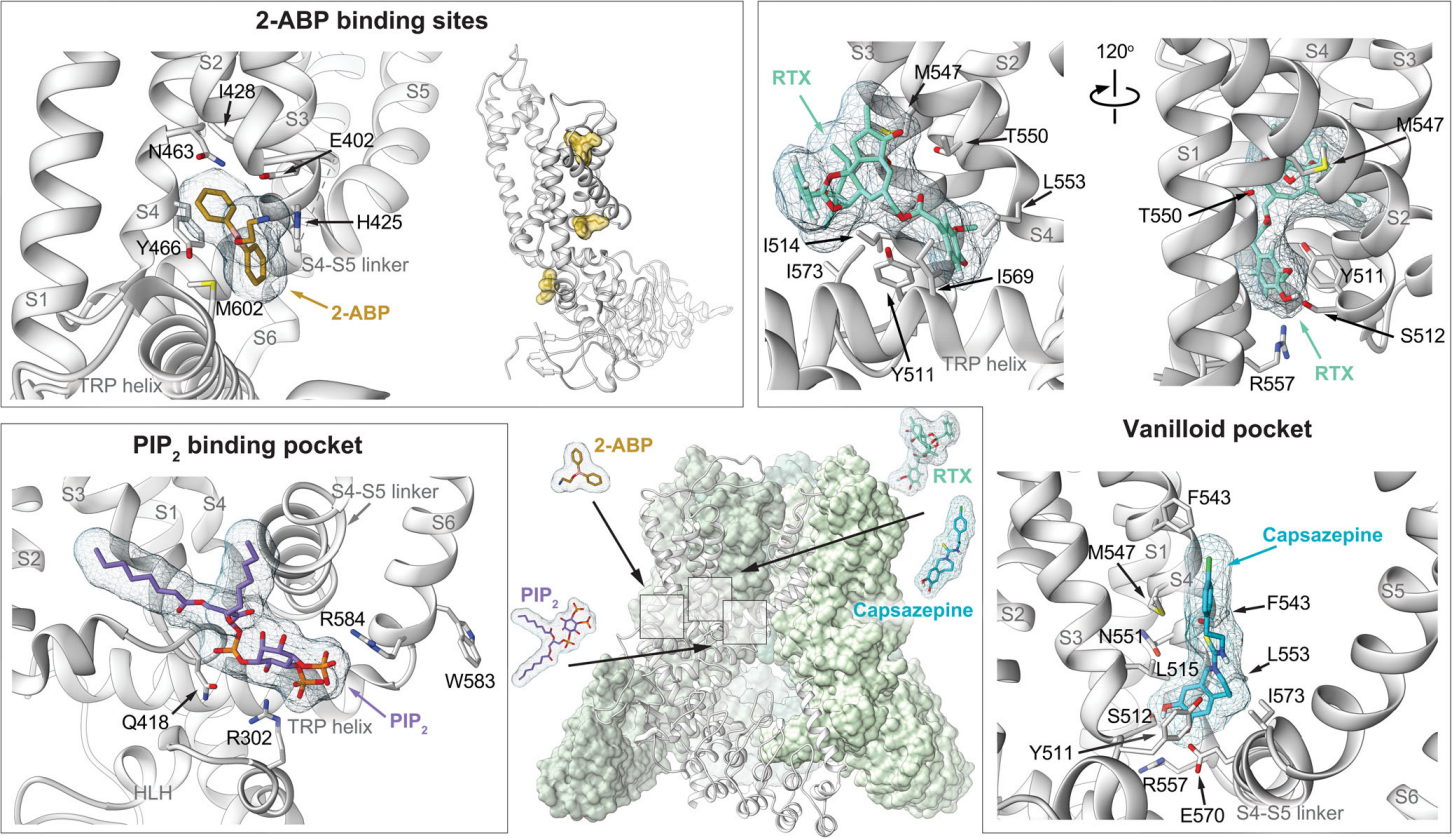
Ligand binding pocket in transient receptor potential vanilloid (TRPV) channels. Surface representation of a TRPV5 channel (green, PDB: 6O1P) with a cutaway view of a single subunit, depicted as the white ribbon (middle). The locations of common TRPV ligand binding pockets are highlighted on the channel surface (rectangles). These pockets that are highlighted in the surrounding boxes as detailed views. On the left top, the *2‐ABP pocket* that is in close proximity to the vanilloid pocket is shown, with TRPV3 depicted as a white ribbon (PDB: 6DVZ). 2‐ABP is depicted in orange, surrounded by a mesh that represents the solvent inaccessible surface. The side chains of interacting residues with 2‐ABP are shown. Next to this, a single TRPV3 subunit is depicted, bound by three 2‐ABP molecules (orange stick diagrams with yellow surface representation). The right panel shows ligand binding in the TRPV1 *vanilloid pocket*. The top two images represent two different views of the ultrapotent vanilloid agonist RTX (teal with mesh surface representation) bound to the vanilloid binding pocket of TRPV1 (white ribbon, PDB: 5IRX). Side chains of residues that are important for RTX binding are highlighted. The bottom image depicts binding of the TRPV1 antagonist capsazepine (light blue, PDB: 5IS0) to the vanilloid binding pocket. At the left bottom, the *PIP*_*2*_ *binding pocket* in TRPV5 (PDB: 6DMU) is shown, with PIP_2_ in violet. The negatively charged head group of PIP_2_ is coordinated by several positively charged residues (R302 and R584)

The TRPV1 structure by Cao et al. first revealed the vanilloid binding pocket (Figure 3), which is located between the S4–S5 linker and S3 helix of one subunit and the S6 helix of another subunit.^7^ In TRPV1, several (ant)agonists (RTX, capsaicin, and capsazepine) were shown to bind to this pocket.^7^, ^23^ Without exogenous ligand, this pocket is occupied by a phosphatidylinositol. Subsequent studies investigating ligand‐dependent gating have significantly benefited from this structural work. A combination of docking and simulation studies, as well as molecular dynamics and thermodynamic mutant cycle analysis were performed to deduce capsaicin's binding mode, reviewed by Yang et al.^40^

Since all TRPV channels share a highly homologous architecture, a similar pocket also exists in TRPV channels even though they are not sensitive to TRPV1‐specific ligands like RTX and capsaicin.^17^, ^18^, ^21^, ^22^, ^36^ It was suggested that the difference in ligand sensitivity is due to the presence of a specific hydrophobic residue in the pocket (F425 in rabbit TRPV5, F470 in rabbit TRPV2).^21^ TRPV1 holds a serine at the same position. In the absence of an exogenous ligand, this pocket is often seen being occupied by a lipid. Of note, while it was originally thought that this pocket would be occupied by a phosphatidylinositol, based on the structure of TRPV1, none of the densities observed in other TRPV channel structures correspond to a phosphatidylinositol lipid. In general, this pocket seems to act as a site of competitive binding since it can accommodate either a ligand or a resident lipid. Displacement of the resident lipid was speculated to regulate channel gating. In TRPV1–3, the resident lipid is proposed to stabilize the closed state and replacement by an agonist induces opening of the channel.^17^, ^18^, ^19^, ^23^ In contrast, the analogous vanilloid pocket of TRPV5 and TRPV6 contains a resident lipid that is proposed to stabilize the channel in an open conformation.^21^, ^22^ McGoldrick et al. demonstrated that mutation of a lipid‐interacting residue (R470E, human TRPV6) expels the lipid and results in a closed state.^22^ A similar mutation in TRPV1 has been shown to ablate capsaicin stimulation.^41^ In TRPV5, binding of the inhibitor econazole in this pocket was shown to rotate S6 in such a way that the lower gate becomes more restrictive, which closes the channel.^21^ Together, this suggests that the structural coupling of the so‐called vanilloid pocket to channel opening is somewhat conserved in TRPV channels, despite the fact TRPV2–6 are not naturally sensitive to vanilloid molecules. This is also supported by the fact that TRPV2 and TRPV3 can be engineered into vanilloid‐sensitive channels by merely introducing a few point mutations.^20^, ^26^

A second lipid‐binding pocket, which is observed in all TRPV structures apart from TRPV4, is located on the cytoplasmic side of the hourglass‐like S1–S4 domain, just above the TRP helix.^17^, ^19^, ^22^, ^23^, ^36^, ^37^ Similar to the vanilloid binding pocket, this S1–S4 pocket allows competitive binding between ligand and resident lipid. Indeed, it was recently shown to underlie 2‐ABP‐dependent regulation of TRPV3 and TRPV6.^19^, ^35^ A comparison of cryo‐EM TRPV3 structures in the closed‐apo state and the open state, bound by its agonist 2‐ABP, revealed three binding sites for 2‐ABP, one of which was previously found in a high‐throughput mutagenesis screen (Figure 3).^18^, ^19^, ^42^ In contrast to TRPV3, TRPV6 channel activity is actually inhibited by 2‐APB binding.^43^ It has been postulated, based on structural studies of TRPV6 bound by 2‐ABP, that binding of this ligand in the S1‐S4 pocket triggers expulsion of the resident lipid. As a result, a hydrophobic cluster forms that ultimately also displaces the resident lipid in the nearby vanilloid binding pocket. Singh et al. propose that this chain of events, through movement of the S4–S5 linker, induces a π‐to‐α helical register shift in S6 that closes the lower gate.^22^, ^35^ As was mentioned before, the vanilloid pocket resident lipid stabilizes TRPV1–3 in a closed state. Hence, lipid displacement by 2‐APB may also explain activation of TRPV3.^19^ This is consistent with previous observations in TRPV1 where channel opening is accompanied by changes in lipid binding.^23^ Although TRPV1 and TRPV2 have not been resolved in complex with 2‐APB, they might contain a similar binding pocket since mutation of residues in this region (TRPV1‐Y554A and TRPV2‐Y514A) increased the affinity for 2‐APB.^35^ Despite high sequence conservation of an important interacting residue (Y466, rat TRPV6), TRPV4 and TRPV5 lack 2‐APB modulation,^43^ which is postulated to result from tighter binding of this S1–S4 lipid.^35^ Of note, two other 2‐APB binding sites were resolved in TRPV3 (Figure 3).^19^ The 2‐APB binding site close to the S1‐S2 loop was suggested to be essential for channel opening as it was the only new 2‐APB density compared to the closed state structure that already contained 2‐APB at the other two binding pockets.^19^

While several groups have tried, the identity of the resident lipids remains to be conclusively determined. Extensive lipid analysis of purified TRPV channels or structural models with a higher resolution will prove valuable in this effort. Some other nonprotein densities located near the selectivity filter have also been identified in the TRPV4 structure,^10^ as well as two more lipid densities close to the S5‐P‐S6 domain in TRPV6.^22^ Again, the identity and function of these lipids have not yet been characterized.

### 4.2 *CaM regulation*

Besides lipids and chemical compounds, the TRPV channels are also regulated by CaM. In TRPV1, CaM binding is likely involved in the desensitization process that occurs after channel activation.^44^ While TRPV2 undergoes a similar desensitization process,^45^ it does not seem to be coupled to CaM binding, and the functional importance of CaM remains to be established. In contrast to TRPV1 and TRPV2, TRPV3 channel activity increases with repeated stimulation, a phenomenon that seems to be dependent on calcium and CaM.^46^ In TRPV4, CaM plays a role in the calcium‐dependent potentiation and subsequent inactivation of the channel.^47^ Overall, CaM is probably best studied in the calcium‐selective TRPV channels, TRPV5 and TRPV6, where it is known to mediate calcium‐dependent channel inactivation.^48^, ^49^

**FIGURE 4:**
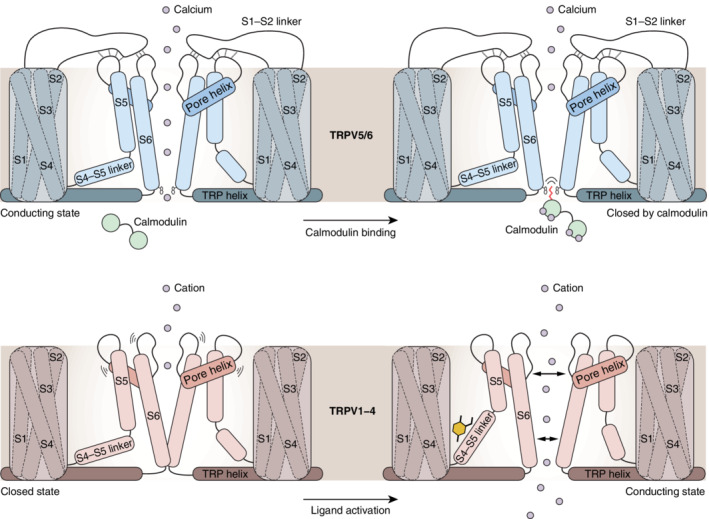
Transient receptor potential vanilloid (TRPV) channel gating models. (a) Schematic of calmodulin‐dependent inhibition in TRPV5 and TRPV6. In the left panel, the channel is unbound by calmodulin and is in an open and conducting state, which allows calcium to traverse the lipid bilayer (beige shade). As a result, the calcium concentration at the intracellular mouth of the channel increases. Calmodulin binds calcium and undergoes a conformational change that lets it insert a lysine (K115) into the TRPV5/6 pore, where it is coordinated by the side chains of W583 (right panel). The insertion of K115 forms a barrier for incoming calcium ions, effectively halting conductance. In both panels, a web of interactions (dotted lines) is highlighted that connects the S1‐S2 linker with the pore helix linkers (both thick lines). This TRPV5/6‐specific feature may be required to restrict movement of the upper gate, which ensures that calcium selectivity remains high, regardless of conformation. (b) Schematic of ligand‐dependent activation of TRPV1–4. The thermosensitive TRPV channels are in a nonconducting state in the absence of a stimulus (left panel). Ligand binding to the channel surface triggers a conformational change in the channel pore that allows the channel to enter a conducting state. The transition towards an open conformation is accompanied by movements in the upper pore region (specifically in TRPV1 and TRPV2), the S4–S5 linker, and movement in S6. Of note, a ligand is depicted in the vanilloid pocket in this schematic (yellow), but binding of ligands to other binding pockets may also trigger the transition from a nonconducting to a conducting state

Three independent groups have recently reported TRPV5 and TRPV6 structures in complex with CaM.^34^, ^36^, ^37^ In line with previous NMR studies,^50^, ^51^ CaM binds to two binding motifs in the C terminus. CaM consists of two lobes (N‐ and C‐lobe) that each contain two calcium binding sites. One full CaM molecule can bind the channel, with the N‐lobe linked to the ARD and a proximal part of the C terminus and the C‐lobe connected to the channel via a distal portion of C terminus. Specifically, a side chain of K115 of the CaM C‐lobe protrudes into the pore, forming a stable cation‐π interaction with W583 of TRPV5/6, thereby blocking the pore (Figure 4).^34^, ^36^, ^37^ Structurally, at least for TRPV5, the channel conformations with or without CaM binding are very similar. Mechanistically, it is suggested that the CaM C‐lobe is constitutively bound at basal intracellular calcium levels and N‐lobe binding is induced at elevated calcium levels. Increased calcium levels after channel opening could induce a conformational change of the binding interface, resulting in fast channel blockade.^36^, ^51^ The TRPV6 structure was resolved in an inactivated state, where binding of CaM leads to a transition in which the channel retains the π‐helical conformation, but is not open for ion permeation.^34^ Structural comparison with the open conformation suggested that binding of CaM does not induce noticeable structural rearrangements of the channel, but rather inhibits channel activity by physically blocking the inner pore region.^36^ This is in line with high calcium selectivity at the channel's top and an intricate calcium‐dependent regulation at the intracellular vestibule. Interestingly, Dang et al. also identified a second CaM density that may be occasionally bound to TRPV5.^36^ Future studies should delineate the functional consequences of a flexible interaction.

In addition, it will be interesting to see to what extend TRPV1–4 undergo similar structural CaM‐dependent regulation, since biochemical assays have found multiple common binding sites.^52^ So far, a crystal structure of the TRPV1 C terminus in complex with CaM also suggests C‐terminal binding,^53^ with two CaM lobes wrapped around a distal C‐terminal helix (residues 767–801). Though, the observed antiparallel orientation might result from using a short peptide fragment and no stable complex of full length TRPV1–4 with CaM suitable for structural studies has been generated so far.

## 5 TRPV5 AND TRPV6 GATING IS DRIVEN BY CALMODULIN

In light of all the structural and functional data that has been gathered on the TRPV channels, we propose that there are two separate gating models that describe TRPV channel activity. TRPV1–4 require a physicochemical activator to displace the resident vanilloid lipid, which opens up the channel through concerted movements of the upper and lower channel gates. In fact, many excellent reviews have been published recently that discuss this ligand‐gated model in more detail.^54^, ^55^, ^56^, ^57^, ^58^ However, we believe that TRPV5 and TRPV6 gate in a different manner under physiological condition, one that is completely dependent on calmodulin and that does not feature any major rearrangements of the TRPV5 and TRPV6 pore region. We base this notion on several observations from functional studies and from the published structures of TRPV5 and TRPV6 with calmodulin.

TRPV5 and TRPV6 favor an open conformation under physiological conditions, which can be closed rapidly through a calcium‐dependent feedback loop. Single channel patch clamp experiments in divalent‐free solutions demonstrate that TRPV5 and TRPV6 exhibit continuous flickering between open and closed states. De Groot et al. have shown previously that parathyroid hormone signaling increases the open probability of TRPV5 and that this is due to a reduction in resident calmodulin binding to the C terminus.^49^ More recently, it has been shown that mutation of W583 at the bottom of the TRPV5 pore results in increased basal cellular calcium levels, cell death and a trend towards an increased open probability, all suggestive of an overactive TRPV5 channel.^59^ The recent structures of TRPV5 and TRPV6, in complex with calmodulin, provide a rationale for these observations and show that calmodulin requires W583 to bind and occludes the TRPV5 and TRPV6 pore. In addition, the structure of a mutated TRPV5 channel, in which the tryptophan residue has been mutated into an alanine (W583A), shows that this residue is essential for stability of the lower pore architecture. In the absence of the tryptophan ring, the lower pore region of TRPV5 adopts an open conformation.^36^ Taken together, these studies suggest that TRPV5 and TRPV6 are ion channels with a remarkably stable baseline open conformation and that they are periodically closed via calmodulin‐dependent pore occlusion to regulate their activity and prevent calcium‐induced toxicity and cell death.

We also deem it unlikely that TRPV5 and TRPV6 are activated by allosteric modulators, besides those that disrupt calmodulin binding. Indeed, to our knowledge, there are no true chemical activators of TRPV5 and TRPV6 described in literature. Of note, there are some TRPV5 and TRPV6 stabilizers, that help to maintain the stable baseline open conformation of the channels and prevent pore collapse, such as phosphatidylinositol 4,5‐bisphosphate (PIP_2_).

## 6 CONCLUSIONS AND FUTURE PERSPECTIVES

The recent “resolution revolution” has significantly improved our understanding of the TRPV channel family. Analysis of the structures allowed for comparison of different family members, both revealing similarities between the channels and explaining their functional differences. It has now provided us with the first steps towards the dynamics behind channel gating and how ligands and lipids play a role in this process. Still, one needs to stay critical on variation in sample preparation, chosen technique (cryo‐EM vs. X‐ray crystallography), data processing or the use of orthologues and truncated proteins as differences can originate from small single amino acid changes. This also highlights how detailed examination of the available data may further reveal which differences between the channels contribute to their biological distinct roles. It has already been shown that thorough analysis of cryo‐EM data can provide valuable insights into channel gating and accompanying structural states, as demonstrated in recent studies by Zubcevic et al.^27^, ^60^ Moreover, complementary use of X‐ray structures can determine which of the pore‐lining amino acids coordinate cations and may allow us to compare the position of differently charged cations to understand selectivity better. This has recently been done for TRPV2, TRPV4, and TRPV6.^10^, ^11^, ^25^ Combining such structural work with detailed functional analysis will deepen our understanding of the channel's behavior and move towards comprehensive structure–function relationships of this interesting TRP subfamily. Ultimately, extrapolation of knowledge towards the complete TRP family will aid in elucidating the differences between the subfamilies and contribute to the understanding of their evolutionary path.

Another major opportunity for future work lies within the field of pathophysiology and pharmacology. Since all channels are associated to disease, improved understanding of the channels' structure–function relationship is key for rationalized design of TRPV‐targeting drugs. The first efforts to use the available structural data to solve these problems have been undertaken. For TRPV1, natural and synthetic small molecules related to capsaicin, have been tested for their anti‐nociceptive effect. Docking studies revealed the importance of interactions between the compounds' amide group and the channel for anti‐nociceptive activity.^61^ The structural models are also used to understand and improve TRPV1 as an anesthetic target. Molecular dynamic studies with 40 different versions of common anesthetics revealed multiple binding sites and predicted interactions that can be used in the screening for new drugs.^62^ Additional studies have explored peptide–channel interactions (i.e., the sea anemone's peptide toxin HCRG21) as well as binding capacities of synthetic derivatives of various agonists including N‐acyl dopamine, capsaicin, RTX, shogaol, and gingerol.^63^, ^64^, ^65^ Moreover, potent new antagonists for TRPV2 were generated by systematically elongating the hydrophobic chain of capsaicin based on the TRPV1 vanilloid binding pocket. Unfortunately, these compounds also activated TRPV1 and are thus still not specific.^66^ Although these first explorative studies have shown the usefulness in drug design, the current structures are not of a high enough resolution to genuinely push this field forward.

Besides drug discovery, the structural maps can also be used as a framework for the analysis of structural and functional consequences of many disease mutations. This is especially relevant for TRPV4 where one mutation can result in several pathologies. Here, segregation of disease‐specific mutations into separate structural regions already provided insight into the putative pathogenic effect of identified mutations.^10^ Moreover, molecular dynamics simulations have examined the potential harmful effect of *TRPV5* genetic variants on TRPV5 structure and function, in relation to their association with kidney stone formation.^67^, ^68^ Less functional TRPV5 channels could decrease calcium reabsorption in the kidney, which may cause hypercalciuria, a risk factor for kidney stones. Additionally, several *TRPV6* mutations have been investigated with in silico mutagenesis and found to play a role channel stability, thereby potentially providing an explanation for disease pathogenesis.^69^, ^70^

Taken together, we have entered a structural era that significantly improved the fundamental understanding of TRPV channels and will likely lead to many more mechanistic insights essential for a full comprehension of TRPV biology.

## CONFLICT OF INTEREST

The authors declare no conflicts of interest.

## AUTHOR CONTRIBUTIONS

**Mark van Goor:** Conceptualization; visualization; writing‐original draft; writing‐review and editing. **Leanne de Jager:** Conceptualization; visualization; writing‐original draft; writing‐review and editing. **Yifan Cheng:** Conceptualization; funding acquisition; supervision; writing‐review and editing. **Jenny van der Wijst:** Conceptualization; funding acquisition; supervision; visualization; writing‐original draft; writing‐review and editing.

## References

[1] Clapham DE , Runnels LW , Strübing C . The TRP ion channel family. Nat Rev Neurosci. 2001;2:387–396. 11389472 10.1038/35077544

[2] Clapham DE . TRP channels as cellular sensors. Nature. 2003;426:517–524. 14654832 10.1038/nature02196

[3] Venkatachalam K , Montell C . TRP channels. Annu Rev Biochem. 2007;76:387–417. 17579562 10.1146/annurev.biochem.75.103004.142819 PMC4196875

[4] Smani T , Shapovalov G , Skryma R , Prevarskaya N , Rosado JA . Functional and physiopathological implications of TRP channels. Biochim Biophys Acta. 2015;1853:1772–1782. 25937071 10.1016/j.bbamcr.2015.04.016

[5] Nilius B , Owsianik G , Voets T , Peters JA . Transient receptor potential cation channels in disease. Physiol Rev. 2007;87:165–217. 17237345 10.1152/physrev.00021.2006

[6] Nilius B , Szallasi A . Transient receptor potential channels as drug targets: From the science of basic research to the art of medicine. Pharmacol Rev. 2014;66:676–814. 24951385 10.1124/pr.113.008268

[7] Cao E , Liao M , Cheng Y , Julius D . TRPV1 structures in distinct conformations reveal activation mechanisms. Nature. 2013;504:113–118. 24305161 10.1038/nature12823 PMC4023639

[8] Liao M , Cao E , Julius D , Cheng Y . Structure of the TRPV1 ion channel determined by electron cryo‐microscopy. Nature. 2013;504:107–112. 24305160 10.1038/nature12822 PMC4078027

[9] Cheng Y . Single‐particle cryo‐EM—How did it get here and where will it go. Science. 2018;361:876–880. 30166484 10.1126/science.aat4346 PMC6460916

[10] Deng Z , Paknejad N , Maksaev G , et al. Cryo‐EM and X‐ray structures of TRPV4 reveal insight into ion permeation and gating mechanisms. Nat Struct Mol Biol. 2018;25:252–260. 29483651 10.1038/s41594-018-0037-5 PMC6252174

[11] Saotome K , Singh AK , Yelshanskaya MV , Sobolevsky AI . Crystal structure of the epithelial calcium channel TRPV6. Nature. 2016;534:506–511. 27296226 10.1038/nature17975 PMC4919205

[12] van Goor MKC , Hoenderop JGJ , van der Wijst J . TRP channels in calcium homeostasis: From hormonal control to structure‐function relationship of TRPV5 and TRPV6. Biochim Biophys Acta. 2017;1864:883–893. 10.1016/j.bbamcr.2016.11.027 27913205

[13] Vennekens R , Hoenderop JGJ , Prenen J , et al. Permeation and gating properties of the novel epithelial Ca^2+^ channel. J Biol Chem. 2000;275:3963–3969. 10660551 10.1074/jbc.275.6.3963

[14] Nilius B , Prenen J , Vennekens R , Hoenderop JGJ , Bindels RJM , Droogmans G . Modulation of the epithelial calcium channel, ECaC, by intracellular Ca^2+^ . Cell Calcium. 2001;29:417–428. 11352507 10.1054/ceca.2001.0201

[15] Nilius B , Weidema F , Prenen J , et al. The carboxyl terminus of the epithelial Ca(2+) channel ECaC1 is involved in Ca(2+)‐dependent inactivation. Eur J Physiol. 2003;445:584–588. 10.1007/s00424-002-0923-9 12634930

[16] Huynh KW , Cohen MR , Jiang J , et al. Structure of the full‐length TRPV2 channel by cryo‐EM. Nat Commun. 2016;7:11130. 27021073 10.1038/ncomms11130 PMC4820614

[17] Zubcevic L , Herzik MA Jr , Chung BC , Liu Z , Lander GC , Lee S‐Y . Cryo‐electron microscopy structure of the TRPV2 ion channel. Nat Struct Mol Biol. 2016;23:180–186. 26779611 10.1038/nsmb.3159 PMC4876856

[18] Zubcevic L , Herzik MA , Wu M , et al. Conformational ensemble of the human TRPV3 ion channel. Nat Commun. 2018;9:4773. 30429472 10.1038/s41467-018-07117-w PMC6235889

[19] Singh AK , McGoldrick LL , Sobolevsky AI . Structure and gating mechanism of the transient receptor potential channel TRPV3. Nat Struct Mol Biol. 2018;25:805–813. 30127359 10.1038/s41594-018-0108-7 PMC6128766

[20] Zhang F , Swartz KJ , Jara‐Oseguera A . Conserved allosteric pathways for activation of TRPV3 revealed through engineering vanilloid‐sensitivity. Elife. 2019;8:e42756. 30644819 10.7554/eLife.42756 PMC6333442

[21] Hughes TET , Lodowski DT , Huynh KW , et al. Structural basis of TRPV5 channel inhibition by econazole revealed by cryo‐EM. Nat Struct Mol Biol. 2018;25:53–60. 29323279 10.1038/s41594-017-0009-1 PMC5951624

[22] McGoldrick LL , Singh AK , Saotome K , et al. Opening of the human epithelial calcium channel TRPV6. Nature. 2017;553:233. 29258289 10.1038/nature25182 PMC5854407

[23] Gao Y , Cao E , Julius D , Cheng Y . TRPV1 structures in nanodiscs reveal mechanisms of ligand and lipid action. Nature. 2016;534:347–351. 27281200 10.1038/nature17964 PMC4911334

[24] Yang F , Vu S , Yarov‐Yarovoy V , Zheng J . Rational design and validation of a vanilloid‐sensitive TRPV2 ion channel. Proc Natl Acad Sci U S A. 2016;113:E3657–E3666. 27298359 10.1073/pnas.1604180113 PMC4932969

[25] Zubcevic L , Le S , Yang H , Lee S‐Y . Conformational plasticity in the selectivity filter of the TRPV2 ion channel. Nat Struct Mol Biol. 2018;25:405–415. 29728656 10.1038/s41594-018-0059-z PMC6025827

[26] Zhang F , Hanson SM , Jara‐Oseguera A , et al. Engineering vanilloid‐sensitivity into the rat TRPV2 channel. Elife. 2016;5:e16409. 27177419 10.7554/eLife.16409 PMC4907692

[27] Zubcevic L , Hsu AL , Borgnia MJ , Lee S‐Y . Symmetry transitions during gating of the TRPV2 ion channel in lipid membranes. Elife. 2019;8:e45779. 31090543 10.7554/eLife.45779 PMC6544438

[28] Chung M‐K , Güler AD , Caterina MJ . TRPV1 shows dynamic ionic selectivity during agonist stimulation. Nat Neurosci. 2008;11:555–564. 18391945 10.1038/nn.2102

[29] Ferreira LGB , Faria RX . TRPing on the pore phenomenon: What do we know about transient receptor potential ion channel‐related pore dilation up to now? J Bioenerg Biomembr. 2016;48:1–12. 26728159 10.1007/s10863-015-9634-8

[30] Chugunov AO , Volynsky PE , Krylov NA , Nolde DE , Efremov RG . Temperature‐sensitive gating of TRPV1 channel as probed by atomistic simulations of its trans‐ and juxtamembrane domains. Sci Rep. 2016;6:33112. 27612191 10.1038/srep33112 PMC5017144

[31] Darre L , Furini S , Domene C . Permeation and dynamics of an open‐activated TRPV1 channel. J Mol Biol. 2015;427:537–549. 25479373 10.1016/j.jmb.2014.11.016

[32] Pan X , Li Z , Zhou Q , et al. Structure of the human voltage‐gated sodium channel Na_v_ 1.4 in complex with β1. Science. 2018;362:eaau2486. 30190309 10.1126/science.aau2486

[33] Shen H , Li Z , Jiang Y , et al. Structural basis for the modulation of voltage‐gated sodium channels by animal toxins. Science. 2018;362:eaau2596. 30049784 10.1126/science.aau2596

[34] Singh AK , McGoldrick LL , Twomey EC , Sobolevsky AI . Mechanism of calmodulin inactivation of the calcium‐selective TRP channel TRPV6. Sci Adv. 2018;4:eaau6088. 30116787 10.1126/sciadv.aau6088 PMC6093632

[35] Singh AK , Saotome K , McGoldrick LL , Sobolevsky AI . Structural bases of TRP channel TRPV6 allosteric modulation by 2‐APB. Nat Commun. 2018;9:2465. 29941865 10.1038/s41467-018-04828-y PMC6018633

[36] Dang S , van Goor MK , Asarnow D , et al. Structural insight into TRPV5 channel function and modulation. Proc Natl Acad Sci U S A. 2019;116:8869–8878. 30975749 10.1073/pnas.1820323116 PMC6500171

[37] Hughes TET , Pumroy RA , Yazici AT , et al. Structural insights on TRPV5 gating by endogenous modulators. Nat Commun. 2018;9:4198. 30305626 10.1038/s41467-018-06753-6 PMC6179994

[38] Kim DM , Nimigean CM . Voltage‐gated potassium channels: A structural examination of selectivity and gating. Cold Spring Harb Perspect Biol. 2016;8:a029231. 27141052 10.1101/cshperspect.a029231 PMC4852806

[39] Palovcak E , Delemotte L , Klein ML , Carnevale V . Comparative sequence analysis suggests a conserved gating mechanism for TRP channels. J Gen Physiol. 2015;146:37–50. 26078053 10.1085/jgp.201411329 PMC4485022

[40] Yang F , Zheng J . Understand spiciness: Mechanism of TRPV1 channel activation by capsaicin. Protein Cell. 2017;8:169–177. 28044278 10.1007/s13238-016-0353-7 PMC5326624

[41] Boukalova S , Marsakova L , Teisinger J , Vlachova V . Conserved residues within the putative S4‐S5 region serve distinct functions among thermosensitive vanilloid transient receptor potential (TRPV) channels. J Biol Chem. 2010;285:41455–41462. 21044960 10.1074/jbc.M110.145466 PMC3009871

[42] Hu H , Grandl J , Bandell M , Petrus M , Patapoutian A . Two amino acid residues determine 2‐APB sensitivity of the ion channels TRPV3 and TRPV4. Proc Natl Acad Sci U S A. 2009;106:1626–1631. 19164517 10.1073/pnas.0812209106 PMC2635798

[43] Hu H‐Z , Gu Q , Wang C , et al. 2‐Aminoethoxydiphenyl borate is a common activator of TRPV1, TRPV2, and TRPV3. J Biol Chem. 2004;279:35741–35748. 15194687 10.1074/jbc.M404164200

[44] Numazaki M , Tominaga T , Takeuchi K , Murayama N , Toyooka H , Tominaga M . Structural determinant of TRPV1 desensitization interacts with calmodulin. Proc Natl Acad Sci U S A. 2003;100:8002–8006. 12808128 10.1073/pnas.1337252100 PMC164702

[45] Mercado J , Gordon‐Shaag A , Zagotta WN , Gordon SE . Ca^2+^‐dependent desensitization of TRPV2 channels is mediated by hydrolysis of phosphatidylinositol 4,5‐bisphosphate. J Neurosci. 2010;30:13338–13347. 20926660 10.1523/JNEUROSCI.2108-10.2010 PMC3001133

[46] Xiao R , Tang J , Wang C , Colton CK , Tian J , Zhu MX . Calcium plays a central role in the sensitization of TRPV3 channel to repetitive stimulations. J Biol Chem. 2008;283:6162–6174. 18178557 10.1074/jbc.M706535200 PMC2287377

[47] Strotmann R , Schultz G , Plant TD . Ca^2+^‐dependent potentiation of the nonselective cation channel TRPV4 is mediated by a C‐terminal calmodulin binding site. J Biol Chem. 2003;278:26541–26549. 12724311 10.1074/jbc.M302590200

[48] Lambers TT , Weidema AF , Nilius B , Hoenderop JGJ , Bindels RJM . Regulation of the mouse epithelial Ca^2+^ channel TRPV6 by the Ca^2+^‐sensor calmodulin. J Biol Chem. 2004;279:28855–28861. 15123711 10.1074/jbc.M313637200

[49] de Groot T , Kovalevskaya NV , Verkaart S , et al. Molecular mechanisms of calmodulin action on TRPV5 and modulation by parathyroid hormone. Mol Cell Biol. 2011;31:2845–2853. 21576356 10.1128/MCB.01319-10 PMC3133394

[50] Bokhovchuk FM , Bate N , Kovalevskaya NV , Goult BT , Spronk CAEM , Vuister GW . The structural basis of calcium‐dependent inactivation of the transient receptor potential vanilloid 5 channel. Biochemistry. 2018;57:2623–2635. 29584409 10.1021/acs.biochem.7b01287

[51] Bate N , Caves RE , Skinner SP , et al. A novel mechanism for calmodulin‐dependent inactivation of transient receptor potential vanilloid 6. Biochemistry. 2018;57:2611–2622. 29505720 10.1021/acs.biochem.7b01286

[52] Zhu MX . Multiple roles of calmodulin and other Ca^2+^‐binding proteins in the functional regulation of TRP channels. Pflugers Arch. 2005;451:105–115. 15924238 10.1007/s00424-005-1427-1

[53] Lau S‐Y , Procko E , Gaudet R . Distinct properties of Ca^2+^‐calmodulin binding to N‐ and C‐terminal regulatory regions of the TRPV1 channel. J Gen Physiol. 2012;140:541–555. 23109716 10.1085/jgp.201210810 PMC3483115

[54] Zubcevic L , Lee S‐Y . The role of pi‐helices in TRP channel gating. Curr Opin Struct Biol. 2019;58:314–323. 31378426 10.1016/j.sbi.2019.06.011 PMC6778516

[55] Pumroy RA , Fluck EC 3rd , Ahmed T , Moiseenkova‐Bell VY . Structural insights into the gating mechanisms of TRPV channels. Cell Calcium. 2020;87:102168. 32004816 10.1016/j.ceca.2020.102168 PMC7153993

[56] Cao E . Structural mechanisms of transient receptor potential ion channels. J Gen Physiol. 2020;152.3. 10.1085/jgp.201811998 PMC7054860 31972006

[57] Hilton JK , Kim M , Van Horn WD . Structural and evolutionary insights point to allosteric regulation of TRP ion channels. Acc Chem Res. 2019;52:1643–1652. 31149807 10.1021/acs.accounts.9b00075 PMC8628317

[58] Yuan P . Structural biology of thermoTRPV channels. Cell Calcium. 2019;84:102106. 31726322 10.1016/j.ceca.2019.102106 PMC6893863

[59] van der Wijst J , Leunissen EH , Blanchard MG , et al. A gate hinge controls the epithelial calcium channel TRPV5. Sci Rep. 2017;7:45489. 28374795 10.1038/srep45489 PMC5379628

[60] Zubcevic L , Borschel WF , Hsu AL , Borgnia MJ , Lee SY . Regulatory switch at the cytoplasmic interface controls TRPV channel gating. Elife. 2019;8:e47746. 31070581 10.7554/eLife.47746 PMC6538378

[61] Rosa‐Lugo V , Acevedo‐Quiroz M , Déciga‐Campos M , Rios MY . Antinociceptive effect of natural and synthetic alkamides involves TRPV1 receptors. J Pharm Pharmacol. 2017;69:884–895. 28374409 10.1111/jphp.12721

[62] Jorgensen C , Domene C . Location and character of volatile general anesthetics binding sites in the transmembrane domain of TRPV1. Mol Pharmaceutics. 2018;15:3920–3930. 10.1021/acs.molpharmaceut.8b00381 30067911

[63] Monastyrnaya M , Peigneur S , Zelepuga E , et al. Kunitz‐type peptide HCRG21 from the sea anemone *Heteractis crispa* is a full antagonist of the TRPV1 receptor. Mar Drugs. 2016;14:229. 10.3390/md14120229 PMC5192466 27983679

[64] Pallavi P , Pretze M , Caballero J , et al. Analyses of synthetic N‐acyl dopamine derivatives revealing different structural requirements for their anti‐inflammatory and transient‐receptor‐potential‐channel‐of‐the‐vanilloid‐receptor‐subfamily‐subtype‐1 (TRPV1)‐activating properties. J Med Chem. 2018;61:3126–3137. 29543451 10.1021/acs.jmedchem.8b00156

[65] Ohbuchi K , Mori Y , Ogawa K , Warabi E , Yamamoto M , Hirokawa T . Detailed analysis of the binding mode of vanilloids to transient receptor potential vanilloid type I (TRPV1) by a mutational and computational study. PLoS One. 2016;11:e0162543. 27606946 10.1371/journal.pone.0162543 PMC5015962

[66] Schiano Moriello A , López Chinarro S , Novo Fernández O , et al. Elongation of the hydrophobic chain as a molecular switch: Discovery of capsaicin derivatives and endogenous lipids as potent transient receptor potential vanilloid channel 2 antagonists. J Med Chem. 2018;61:8255–8281. 30176215 10.1021/acs.jmedchem.8b00734

[67] Wang L , Holmes RP , Peng J‐B . The L530R variation associated with recurrent kidney stones impairs the structure and function of TRPV5. Biochem Biophys Res Commun. 2017;492:362–367. 28847730 10.1016/j.bbrc.2017.08.102 PMC5639706

[68] Wang L , Holmes RP , Peng J‐B . Molecular modeling of the structural and dynamical changes in calcium channel TRPV5 induced by the African‐specific A563T variation. Biochemistry. 2016;55:1254–1264. 26837804 10.1021/acs.biochem.5b00732 PMC5304435

[69] Burren CP , Caswell R , Castle B , et al. TRPV6 compound heterozygous variants result in impaired placental calcium transport and severe undermineralization and dysplasia of the fetal skeleton. Am J Med Genet A. 2018;176:1950–1955. 30144375 10.1002/ajmg.a.40484 PMC6563443

[70] Suzuki Y , Chitayat D , Sawada H , et al. TRPV6 variants interfere with maternal‐fetal calcium transport through the placenta and cause transient neonatal hyperparathyroidism. Am J Hum Genet. 2018;102:1104–1114. 29861107 10.1016/j.ajhg.2018.04.006 PMC5992228

